# Downregulation of Blood Monocyte HLA-DR in ICU Patients Is Also Present in Bone Marrow Cells

**DOI:** 10.1371/journal.pone.0164489

**Published:** 2016-11-28

**Authors:** Valérie Faivre, Anne-Claire Lukaszewicz, Didier Payen

**Affiliations:** 1 Inserm, U 1160, Paris, France; 2 University Paris Diderot, Sorbonne Paris Cité, Paris, France; 3 AP-HP, Hôpital Lariboisière, Département d’Anesthésie - Réanimation - SMUR, Paris, France; Agency for Science, Technology and Research - Singapore Immunology Network, SINGAPORE

## Abstract

**Background:**

The downregulation of blood monocyte HLA-DR expression also occurs in tissue infiltrative cells in a context of acute clinical inflammation, especially sepsis. This context favors the development of secondary infections and results from various mechanisms. Little is known about HLA-DR expression on bone marrow (BM) cells of the monocyte lineage, the source of circulating monocytes. This study analyzed the BM HLA-DR expression in ICU patients compared to BM monocytes from non-ICU patients and to blood monocytes of control healthy donors. A potential dysfunction of myeloid differentiation was investigated in a sub-population of these ICU patients to characterize the phenotype of the immature forms of monocytes and granulocytes in BM.

**Methods and Findings:**

BM and blood were drawn from 33 ICU and 9 non-ICU patients having a BM analysis to precise the etiology of abnormal low count in blood cells. The data were compared with blood cells of 28 control donors. Flow cytometry was used for both HLA-DR expression and phenotyping of immature forms of monocytes and granulocytes. HLA-DR expression was downregulated in both blood and BM monocyte in ICU patients compared to BM of non-ICU patients and blood of control donors. Amplitude of HLA-DR downregulation was comparable in septic and non-septic ICU patients. The phenotype of immature forms of monocytes and granulocytes in BM (n = 11) did not show abnormal myeloid (monocyte + granulocyte) differentiation.

**Conclusion:**

The downregulation of HLA-DR in BM monocyte lineage is present in ICU patients without major changes in myeloid cells. It may result from a regulation mediated by soluble and/or neuro-endocrine factors present in BM cell microenvironment.

## Introduction

A paradigm shift for sepsis involves the evolution of the blood immune status from a state of initial hyperactivity to early downregulation and immunodepression [1]. This immunodepression has been shown to involve both innate and adaptive immunity [2, 3] in circulating and tissue monocytes and lymphocytes [4]. This syndrome has mainly been observed in patients with severe sepsis but has also been found in patients with many other life-threatening situations [5, 6]. The immunodepression seen in these patients may explain why almost all randomized clinical trials testing anti-inflammatory treatments have failed to improve outcomes [7]. Immunodepression in patients has been associated with unresolved infections, the reactivation of viral infection, and the occurrence of secondary infections [1], which leads to questions regarding the potential benefit of using immunostimulating drugs [1]. The decision to use these immune-boosting drugs and controlling the effect of these drugs requires monitoring of the immune response. Among the potential parameters to monitor, blood monocyte HLA-DR was the most frequently reported in the blood [6, 8, 9] and tissues [4]. The downregulation of HLA-DR expression results from intricate mechanisms such as the release of IL-10 [10] or cortisol [11].

Because infiltrating immune cells in both blood and tissues can be downregulated, this may also occur in bone marrow cells, which receive peripheral blood containing immunosuppressive factors. Little is known about the expression of HLA-DR in bone marrow cells and how its expression can be modified by central (BM and nervous system) regulation [12–14]. Systemic inflammation induces the stimulation of hematopoietic stem cells (HSC) by ligands of Toll-Like Receptors (TLR), Pattern Recognition Receptors (PRR), and growth factors produced by stimulated endothelial cells. This micro-environment increases myelopoiesis and decreases lymphopoiesis [15–17]. Although hematopoiesis has been investigated in hematological diseases and cancer in humans [18], it has not been described in intensive care patients with acute inflammation. In a burnt mice model [19, 20] it has been demonstrated a hypo-reactivity of developing monocytes and DC in the BM and their subsequent migration to the periphery. This provides a plausible explanation for the immunosuppression after a critical burn injury and sepsis. Considering the myeloid lineages, it appeared important to evaluate also the granulopoiesis seen as a proliferative (promyelocytes + myelocytes) and maturation (metamyelocytes + PMNs) compartments. These cells have been shown to respond to “an emergency granulopoiesis” limited to the early phase of acute injury [21].

Access to BM samples from ICU patients gave us the opportunity to describe the following aspects: 1- the expression of HLA-DR in BM (marker of monocytic differentiation) and blood monocytes in ICU patients *versus* healthy controls and non-ICU patients; 2- CCR2 expression, which is known to increase from unspecialized cells to blood monocytes; and 3- the CD11b and CD62L expression (adhesion molecules) in 4 stages of differentiation in granulocyte lineage.

## Methods

### Patients and controls

The study was approved by the Société de Réanimation de Langue Française Ethics Committee (# CE SRLF 11 369). BM and blood were collected for medical follow-up. Since no additional samples were drawn for the study and cells or plasma were not stored after completion of the experiments, patients or their next of kin received oral information about the study and gave their verbal consent for the use of samples remainders according to the French Ethical Law.

Between June 2007 and March 2014, we prospectively enrolled ICU patients who were 18 years or older with systemic inflammation related to sepsis or not when the senior in charge decided to analyze the BM. The decision was motivated by inexplicably low counts in blood cell population(s) and was validated by an independent hematologist. BM analysis was aimed at diagnosing a macrophage activation syndrome, a toxin-induced BM dysfunction or a metabolic deficit. Patients who were pregnant, had myelodysplasia or were treated with chemotherapy and who had a risk of aplasia were excluded. Sepsis and septic shock were diagnosed and classified according to the ACCP/SCCM consensus criteria [22]. During the same time period, the specialist (internal medicine or rheumatology) determined whether a BM analysis should be conducted for non-ICU patients during medical consults or short hospitalizations. When such BM analysis was normal (n = 9) (conclusion made by a blinded hematologist), the results were used to determine “normal” ranges of HLA-DR expression in BM monocyte. Finally, the normal values for blood parameters were determined using blood samples from 28 healthy volunteers (10 males, 34 [18] y/o).

Blood and BM samples were collected in EDTA tubes for routine examination. The remaining material was used for specific studies with no additional sampling. Because conservation of biological samples was not necessary, we did not require written informed consent, as noted by the Ethical Committee according to the French Ethical Law. The information about the study was given only to the patient and the next of kin, who gave verbal consent for using BM and blood data. Age, gender, time and indication for BM analysis were collected. For ICU patients, the delay between hospitalization and time of arrival in the ICU was also collected.

Bone marrow analysis was blindly conducted by a trained hematologist. Samples were obtained by sternal aspiration and were immediately smeared on glass slides. After May Grünwald Giemsa coloration, analysis was conducted under the microscope using 50 and 100X magnifications to determine the percentage of hemoblasts, granulocytes (including myeloblasts, promyelocytes, myelocytes, metamyelocytes, polymorphonuclear neutrophils (PMNs), polymorphonuclear eosinophils and basophils), lymphocytes, monocytes, erythroblasts (including proerythroblasts, basophilic, polychromatophilic and acidophilic erythroblasts), and other pathological cell populations, if present. Based on these results, the proportions of myeloblasts, promyelocytes, myelocytes, metamyelocytes, PMNs and monocytes were expressed as a percentage of the total leucocyte population.

### Flow cytometry

The flow cytometry analyses were performed with a FACsCanto II instrument (BD Biosciences, San Jose, CA, USA) with FACsDIVA software (BD). The selection of cell populations was based on forward/side scattering and surface molecule expression. Blood monocytes were selected as CD14-positive (pos) cells with intermediate SSC for which HLA-DR expression was analyzed. These blood monocytes were compared to bone marrow CD14 high cells, which were considered to be the most differentiated cells among the monocytic lineage [23]. For the BM monocyte lineage study, the following characteristics were used to differentiate between the different CD45-positive (marker of leucocytes) cell populations: myelo-/monoblasts: SSClow/CD34high; promonocytes: CD34neg/CD33high/CD14neg; monocytes: CD34neg/CD33high/CD14pos. In addition all monocyte sub-populations were further analyzed based on their CD16 expression, in CD16 positive (pos) and CD16 negative (neg) cells.

For the granulocyte lineage, the following characteristics were used to differentiate between the CD45-positive cell populations: promyelocytes + myelocytes (promyelo-myelo): CD16low/ CD66binterm-high/CD13high-low; metamyelocytes: CD16pos/CD66binterm/CD13interm and PMNs: CD16high/CD66blow/CD13high [23, 24]. CD11b was used to gate myelocytes (CD11bneg) and promyelocytes (CD11bpos) concomitantly with the use of low CD16 expression for alignment.

For cell surface marker analysis, 50 μl of blood or bone marrow (EDTA Vacutainer, BD, Le Pont-de-Claix, France) was incubated 30 minutes at room temperature with the appropriate antibodies, in concentrations recommended by manufacturers: anti-CD14-FiTC (clone RMO52), anti-CD3-FiTC (clone UCHT1) (Beckman Coulter Immunotech, Marseille, France), anti-HLA-DR-PE (clone G46-6), anti-CD62L-PE (clone DREG-56), anti-CD16-PerCP-Cy5.5 (clone 3G8), anti CD11b-PE (clone ICRF44)(BD), anti-CCR2-PE (clone 48607) (R&D Systems, Abingdon, UK), anti-CD33-PE-Cy7 (clone WM-53)(Novus Biologicals, Cambridge, UK), anti-CD34-APC (clone AC136)(Miltenyi Biotec, Bergisch Gladbach, Germany), anti-CD13-APC (clone TÜK 1)(Invitrogen Molecular Probes, Camarillo, CA, USA), and anti-CD45-APC-Cy7 (clone HI30) (Biolegend, San Diego, CA, USA). After 10 minutes of red blood cell lysis (FACs Lysing solution, BD), and sample wash with phosphate buffer saline (Life Technologies, Paisley, UK), cells were fixed with 1% solution of paraformaldehyde (Sigma, St. Quentin Fallavier, France) and analyzed within 24 hours.

The expression of HLA-DR, CCR2, CD11b and CD62L was quantified as the number of sites per cell (antibodies bound per cell, AB/C), using a commercial kit (Quantibrite^™^, BD). The linear regression between fluorescence intensity and AB/C was calculated every month and showed good reproducibility over the whole study period, avoiding cytometer variations. PE/antibody ratio was assumed to be stable over time by the use of commercial antibodies. The normal range for blood mHLA-DR expression in healthy donors using our set-up conditions (n = 28; median age, 34 yrs old (range, 18–57); sex ratio, 64% female) was 16884 (5842–29175) AB/C.

### Statistical analysis

The results were expressed as the median and the interquatile range (IQR). Differences between patient groups and healthy donors were tested using the non-parametric Kruskal-Wallis test. When statistical significance was found, inter group differences were tested with the Mann-Whitney test. Differences between bone marrow and blood monocytes were tested using the non-parametric Wilcoxon test. Differences in marker expression between successive stages of differentiation in the granulocyte or monocyte lineage were tested using the non-parametric Friedman test. When statistical significance was found, the Wilcoxon test was used to make paired comparisons between stages.

## Results

### Patients

**Fig 1:**
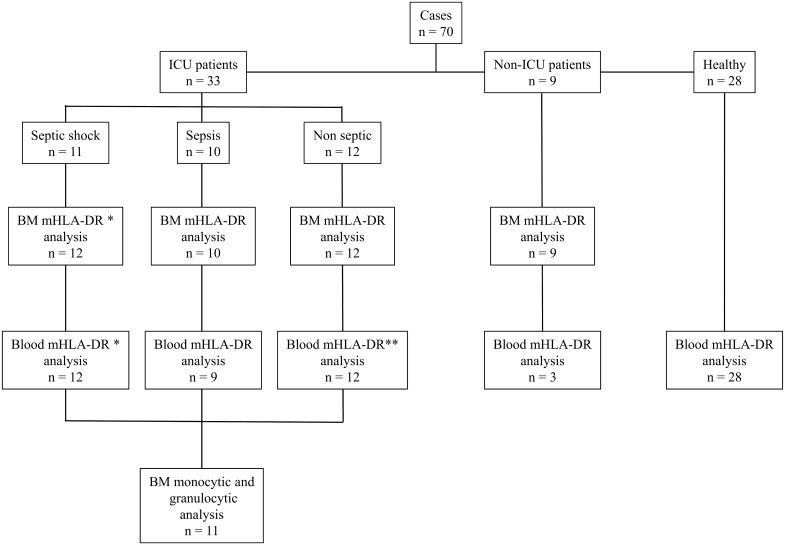
Number of patients and study design.

Fig 1 shows the flowchart of the study. The clinical characteristics of the 42 patients studied are shown in Table 1. ICU patients (individual results in S1 Table) were either in septic shock (n = 11) or did not exhibit septic shock (n = 10 septic; n = 12 non-septic). SAPS II in septic shock patients was higher than in the non-septic shock group and in non-septic patients. The main indication for BM sampling was the presence of significant thrombocytopenia (26/33; 79%), which was not clearly linked to peripheral consumption as observed in DIC. For most patients, the hematologist diagnosed the patients with peripheral thrombocytopenia, along with a diagnosis of MAS for some or hemophagocytosis in septic shock patients or non-septic patients, which is a rare diagnosis in ICU patients. In contrast, the patterns of reactive BM and an excess granulocyte precursors were observed in half of the samples, with no abnormalities and/or interruption at different stages of the monocytic lineage.

**Table 1:** Clinical characteristics of the patients as well as the main diagnosis by the hematologist using BM analysis.

	Septic shock (n = 11 patients, n = 12 samples)	Sepsis (n = 10)	Non-septic (n = 12)	Non-ICU (n = 9)
Age (y/o)	66 [25]	48 [15]*§	64 [26]	72 [24]
Gender (n (%) Male)	6 (55)	6 (60)	6 (50)	6 (67)
Time of ICU admission (days post-hospital admission)	1 [2]	1 [2]	3 [8]	
SAPS II at ICU admission	52 [19.8]	23.5 [13]*#	39.5 [20.5]*	
Time of BM analysis (days post-ICU admission for ICU patients; post-hospital admission for non-ICU patients)	6 [7]	10 [11]	5 [25]	2 [5]
MAS diagnosis (n (%))	2 (17)	0 (0)	2 (17)	0 (0)
Reactive BM (n (%))	6 (50)	6 (60)	4 (33)*	
Excess of granulocyte precursors	5 (42)	5 (50)	3 (25)*	

ICU patients (n = 33, septic shock, sepsis and non-septic), and non-ICU patients (n = 9). Results are expressed as the median and the interquartile range [IQR] or, when indicated, in numbers (n) and percentages (%).

* p<0.05 *vs*. septic shock,

^#^ p<0.05 vs. non-septic,

^§^ p<0.05 *vs*. non-ICU, Mann-Whitney.

### HLA-DR expression in blood monocytes and in bone marrow CD14high monocytes

**Fig 2:**
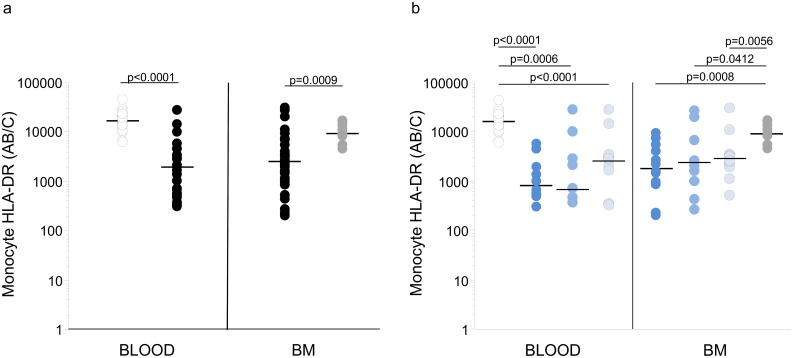
Blood and BM mHLA-DR expression in patients and controls. a/ ICU patients (black), non-ICU patients (grey) and healthy controls (white). Individual values (circles) and the median (line) in AB/C. b/ septic shock ICU patients (dark blue), septic ICU patients (middle blue), non-septic ICU patients (light blue), non-ICU patients (grey) and healthy controls (white). Significant Kruskal-Wallis test led to perform Mann Whitney comparisons. No difference in each group between blood and BM (Wilcoxon test) were observed.

As previously observed, the blood mHLA-DR levels of ICU patients (1976 [2812] AB/C) were dramatically lower than in the control group (p<0.0001) (Fig 2a). Because the monocyte lineage in BM was classified as normal for all patients by the blinded hematologist, the HLA-DR expression of BM monocytes was measured in their most differentiated form, *i*.*e*., cells with the highest expression level of the CD14 surface marker. The BM mHLA-DR levels in ICU patients were also reduced to a level (2510 [3289] AB/C) that did not differ from the mHLA-DR expression in the blood (Fig 2a). This BM mHLA-DR expression in ICU patients was lower than in non-ICU patients (p = 0.009). Among non-ICU patient, 3 patients had a blood mHLA-DR expression level that was similar to the levels of blood mHLA-DR in healthy volunteers (S2 Table).

Fig 2b shows the blood and BM mHLA-DR expression levels in the different subgroups. Compared to healthy controls, the blood mHLA-DR expression levels were reduced in septic shock patients (p<0.0001, Mann-Whitney), in patients with sepsis (p = 0.006), and in non-septic patients (p<0.0001). Regarding BM mHLA-DR, all ICU patients groups were also downregulated compared to non-ICU patients. Interestingly, no difference was observed between the ICU sub-groups for mHLA-DR in the blood or BM (Mann Whitney).

### Analysis of differentiation stages for BM monocytic and granulocytic lineages in ICU patients

The differentiation stages of monocytes and granulocytes were analyzed either by FACs or microscopy (S3 Table). Because of the various criteria used to discriminate between the different stages (protein expression for FACs and morphological elements using microscopy), some differences were observed. To limit these differences, cell populations were grouped according to the closest differentiation stages as follows: myeloblasts and monoblasts were pooled and analyzed as the myelo-/monoblast stage, and promyelocytes and myelocytes were grouped into one promyelo-myelo population. The proportion of monocytes was normal [23]. The percentage of myelo-/monoblasts, which are the most immature forms, was different between FACs (0.283 [0.372]%) and microscopic analysis (1.4 [1.075]%, p = 0.0208, Wilcoxon) but was consistently low. In the granulocyte lineage, a predominance of the promyelo-myelo was observed with FACs analysis (22.8 [24.7]%) but less with microscopy (18.8 [10.75]%). Despite these differences, the hematologist described an excess of this subpopulation in 12 (most severe inflammation assessed by SAPSII; S1 Table) of the 33 ICU patients (S3 Table).

### HLA-DR (n = 10) and CCR2 (n = 11) expression among the different stages of BM monocyte differentiation in ICU patients

**Fig 3:**
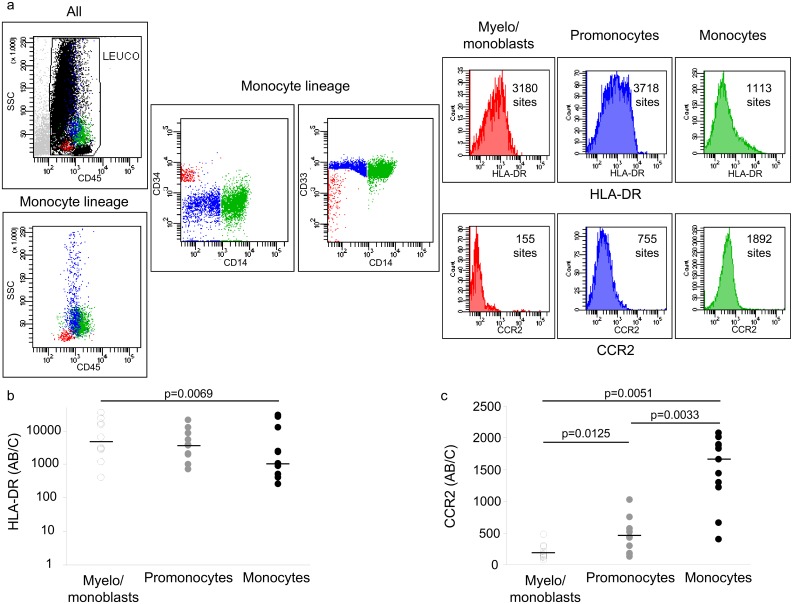
Monocyte differentiation stages in the BM. Monocyte differentiation stages were characterized by CD45, CD34, CD33 and CD14 expression. HLA-DR and CCR2 expression were measured in each stage and expressed as the number of sites per cell. a/ example of FACs analysis for monocyte differentiation (red: myelo/monoblasts, blue: promonocytes, green: monocytes), and the HLA-DR and CCR2 expression levels in each stage (patient 31, see S1 Table); b/ individual values for HLA-DR expression (n = 10); c/ individual values for CCR2 expression (n = 10 for myelo/monoblasts, n = 11 for other cell populations). Wilcoxon comparisons.

Fig 3a shows a typical flow cytometry analysis of the monocyte lineage in BM based on CD45, CD34, CD33 and CD14 expression. The expression of HLA-DR (Fig 3b) and CCR2 (Fig 3c) was measured for each stage of differentiation. If HLA-DR expression was heterogeneous, a significant decrease was observed between the myelo-/monoblast and promonocyte stages (7 of 10 patients) and between the promonocyte and monocyte stages (9/10 patients) (p = 0.0136 Friedman test, p = 0.0069 Wilcoxon test between myelo-/monoblasts and monocytes). At the same time, CCR2 significantly increased between successive stages in all patients (p = 0,0001 Friedman test, p = 0.0125; 0.0033 and 0.0051, Wilcoxon test between myelo-/monoblasts and promonocytes, promonocytes and monocytes, and myelo-/monoblasts and monocytes, respectively).

### CD11b and CD62L (n = 10) expression among the different stages of BM granulocyte differentiation in ICU patients

**Fig 4:**
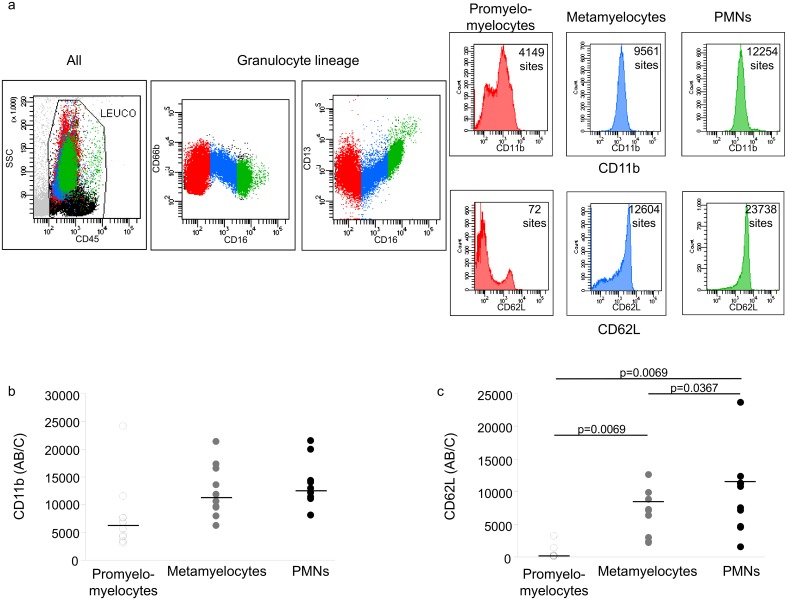
Granulocyte differentiation stages in the BM. Monocytes differentiation stages were characterized by CD45, CD66b, CD13, and CD16 expression. CD11b and CD62L expression were measured in each stage and expressed as the number of sites per cell. a/ example of FACs analysis for granulocyte differentiation (red: promyelo-myelocytes, blue: metamyelocytes, green: PMNs), and the expression of CD11b and CD62L in each stage (patient 30, see S1 Table); b/ individual values for CD11b expression (n = 10); c/ individual values for CD62L expression (n = 10). Wilcoxon comparisons.

Fig 4a shows a typical flow cytometry analysis of the granulocyte lineage in BM based on CD45, CD66b, CD13, and CD16 expression. The expression of CD11b (Fig 4b) and CD62L (Fig 4c) was measured for each stage of differentiation and showed similar patterns. CD11b and CD62L both increased between stages of differentiation, but this variation was significant only for CD62L (Friedman test), especially between promyelo-myelocytes and metamyelocytes (9 of 10 patients, p = 0.0069).

### Analysis of CD16 positive and negative cells in monocyte lineage

**Fig 5:**
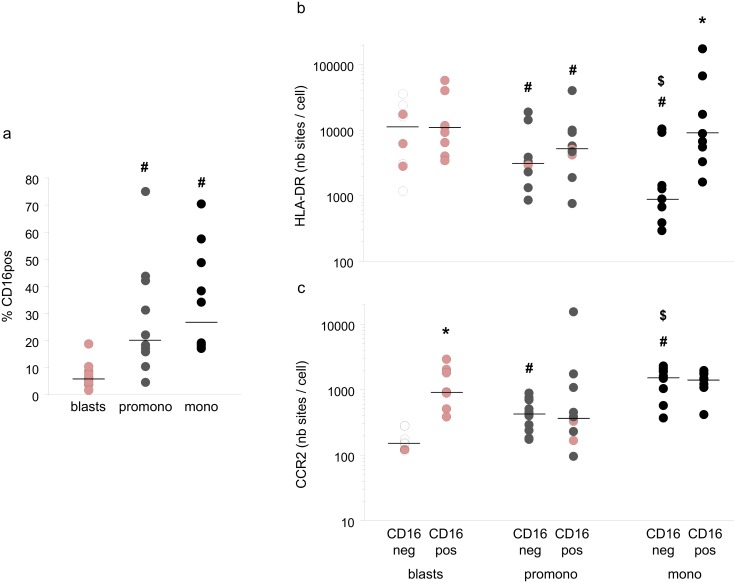
Analysis of CD16 positive and CD16 negative cells in BM monocyte lineage. a/ Percentage of CD16+ cells in blasts, promonocytes and monocytes. b/ HLA-DR expression in CD16neg and CD16pos cells in blasts, promonocytes, and monocytes (number of sites /cell). c/ CCR2 expression in CD16neg and CD16pos cells in blasts, promonocytes, and monocytes (number of sites /cell). Red filled circles corresponded to data obtained in rare events populations. Individual values and median. # p < 0.05 vs blasts p < 0.05 vs blasts, $ p < 0.05 vs promonocytes, * p < 0.05 vs CD16neg, Wilcoxon test.

Since blood monocyte subtypes CD16 positive and CD16 negative have different functions and phenotypes [25], we have investigated CD16 expression in BM monocyte lineage cells. CD16 expression became detectable at the promonocytic stage, being almost undetectable on blasts (S1 Fig). Expression profile allowed to select CD16pos and CD16neg monocytes, but not the intermediate CD16low population normally present in blood. Median percentage of CD16pos cells was 20.2% in promonocytes and 26.8% in monocytes (Fig 5a). We also analyzed HLA-DR and CCR2 expression in CD16pos and CD16neg cells. A significant trend for downregulation of HLA-DR expression was observed along the differentiation in CD16neg (Fig 5b), which was not observed in CD16pos. Conversely, there was no difference in CCR2 expression between CD16pos and CD16neg cells at the promonocyte and monocyte stages.

## Discussion

It is now largely acknowledged that the blood monocyte HLA-DR expression rapidly decreases in the acute phase of severe diseases in ICU patients [9]. Based on these findings and on the observed HLA-DR downregulation in tissue-infiltrating cells [4], HLA-DR expression was accepted as a good marker for innate immunodepression and can be considered a useful marker for immune status monitoring in patients [9]. The mechanisms for downregulation of mHLA-DR in the blood are multifactorial, being under the control of anti-inflammatory mediators such as IL-10 [10], stress hormones such as cortisol [11], a deficit of lymphokines [26], and a failure in cell metabolism [27]. Most of the modulators of mHLA-DR expression that are present in the blood are also present in perfused compartments, including in immune cell reservoirs such as the BM, which is the main source of hematopoiesis. Although the BM contains all forms of blood cells that differentiate into various lineages, including mature cells that are ready to exit into the blood, little is known about HLA-DR expression in BM. The main results of this study are: 1- a similar downregulation of monocyte HLA-DR was present in blood and bone marrow monocyte lineage in different groups of ICU patients, septic or not compared to controls and non-ICU patients; 2- within the myeloid cells, the granulocyte lineage compartments (proliferation vs maturation) were almost normal.

Limits of the study: some limitations preclude the generalization of these results. The number of enrolled patients in ICU was relatively small (n = 33) and concerned only patients for whom abnormal blood cell count had motivated a bone marrow investigation in absence of clear etiologies. These patients may then differ from those without such abnormalities and results on BM cannot be generalized. Etiologies for systemic inflammation in ICU patients were heterogeneous with severe sepsis or septic shock but also brain injury, hemorrhagic or vascular diseases, major surgery and pre-eclampsia. Considering the results, it can be concluded that within the limit of this study, different etiologies for inflammation induce similar modifications in BM cells.

Compared to mice, hematopoiesis is rarely studied in humans especially in ICU patients, except for hematological pathologies. Human BM hematopoietic stem cells (HSC) are known to be multipotent, and oligopotent progenitors of blood cells, expressing the CD34 antigen [23, 28, 29]. The CD34+ cells called blasts, represent 0.7–1% of BM cells [30] under normal conditions, having a moderately bright positive and heterogeneous expression of HLA-DR [31, 32, 33]. Such expression of HLA-DR between promonocyte and monocyte differentiation has not received attention and thus remains unclear [34, 35].

Screening the literature, no *ex vivo* experiments testing the monocyte differentiation from human BM cells in septic context are available. Muthu et al and Howell et al have addressed such issue using a burnt mice model with sepsis, and evaluated the monocyte differentiation [19, 20]. The authors said “burn injury alters the lineage commitment of bone marrow GMPs toward monocyte differentiation and away from DCs.” The differentiated cells exhibited then a reduction in MHC-II expression.

In our study, HLA-DR expression in blasts was heterogeneous but was significantly higher than in mature CD14+ monocytes in BM. This observation has to be considered cautiously because of the limited number of samples and heterogeneity of the results. This finding suggests a downregulation within differentiation steps during acute systemic inflammation in ICU patients, a hypothesis that fits well with the numerous publications reporting the decreased blood monocyte HLA-DR expression. Only 2 patients out of 10 exhibited an increase of HLA-DR along monocytic differentiation steps. The time delay after initial injury for sampling BM was different, and may then correspond to different phases of the inflammatory process [1]. For most of the patients, the delay was long enough to miss the transient increase in HLA-DR expression along monocytic development. It can also be hypothesized that the “tolerant” phenotype might be transient with re-programmation of the cells initial differentiation towards more reactive cells to agonists (cytokine, PAMPs, DAMPs). Such hypothesis had been verified in burnt mice model for measurements after 72 hrs [19, 20].

The progressive increase in CCR2 expression (the receptor to MCP-1) along BM monocyte differentiation also indicates that blood monocytes were mature and ready to respond to a MCP-1 gradient and to exit BM towards blood [36]. These results suggest that the downregulation of HLA-DR expression observed in blood monocytes already occurs in BM leading to an increase in HLA-DR^low^ blood monocytes. In addition, the low expression of HLA-DR in blood monocytes does not support the hypothesis of a premature BM exit with a high or not low HLA-DR expression (Fig 3). Contamination of the BM sample by blood monocytes should be ruled out to avoid the risk of interpreting the BM HLA-DR^low^ monocytes as contamination by blood monocytes. This technical limit can be excluded because the proportion of T cells (CD3+ cells, n = 11) in BM samples was only 4.1%, a clear difference with the proportion observed in blood. Investigation of monocyte subtypes CD16pos and CD16neg demonstrate the absence of CD16 expression in blasts, with an increase in CD16pos at promonocytes and monocytes stages. Only the CD16neg cells had a reduced expression in HLA-DR as observed in blood. CD16pos also called pro-inflammatory were present and continue to constitutively express HLA-DR, even in conditions where HLA-DR is pathologically reduced [37].

The release of immune mediators and neuroendocrine factors in the blood and BM may maintain stable cell phenotypes for several days. Similar observations of low HLA-DR expression in blood or BM in ICU patients at different times of evolution strongly suggests an anti-inflammatory and immune-depressed phenotype. Being under the control of similar molecules, this can be seen as a modulation of inflammation to limit the risk of multiple organ failure [1]. The maturation and release of less activated immune cells in the blood appeared maintained as suggested by the simultaneous increase in CCR2 expression. In a murine peritonitis model, the peak of IL-10 expression in abdominal cavity promotes the differentiation of recruited monocytes towards HLA-DR^low^ macrophages specialized in apoptotic cells elimination. During acute inflammation, such IL-10 expression leads to accumulation at the site of HLA-DR^low^ macrophages. At a later phase, the decrease in local IL-10 concentration promotes monocyte differentiation towards HLA-DR^high^ macrophages and favors their ability to present antigens [38]. Although not been demonstrated in BM, such a mechanism might downregulate monocyte function and their ability to express HLA-DR before exit to the blood compartment being subsequently recruited by different tissues.

The granulocyte lineage in our BM study showed a normal proliferation and maturation proportions. Under normal conditions, Elghetany et al have reported the BM neutrophilic granulocytes (CD15+, CD33+, CD43+, CD66b+) composed of 7±4% of promyelocytes, 27±9.2% of myelocytes, 24±6.1% of metamyelocytes, and 42.2±12.8% of PMNs [39]. Although our results have been obtained using similar population markers, the cell proportion within granulocytic lineage was normal and not increased in maturation compartment (promyelo + myelo = 36%) with a normal proportion of metamyelo (34%) but with a decreased in PMNs proportion (29%). The initial increase in maturation compartment might have been missed in relation of the delay for BM sampling. The low PMNs percentage could be explained by a strong peripheral demand, increasing the exit of differentiated cells from BM but trapped within different organs. CD11b expression can be used as a marker of PMN activation but also as a marker of differentiation. We observed low CD11b expression in most undifferentiated forms in BM (lowest level of CD16 when gating on promyelo+myelo). The expression of both CD11b and CD62L along the stages of differentiation in our study confirmed the findings in the literature showing the absence of changes in cell phenotypes in BM granulocytes.

The observed results are important in the context to the current trials using immune-stimulating drugs as GM-CSF, INFγ, PD-1 antibody or IL-7. The push of immune system is still an opened debate. First, the criteria and biomarkers to guide and monitor “immunotherapy” are a prerequisite. Among the biomarkers, only HLA-DR expression was largely reported with an adequate time response, cost and effectiveness and may represent a major advance [9]. In our practice, the indication to boost the immune system is based on an observed plateau of low level HLA-DR expression associated with persistent infection (initial or secondary infections) despite adequate anti-microbial therapy [6]. The therapeutic potential targets may help to select the adequate molecule to use. The well demonstrated INFγ deficit in sepsis justifies the use of recombinant INFγ targeting mainly innate immunity to restore the monocyte function [40]. The many defects in neutrophil function occurring in sepsis had motivated 2 RCT using recombinant granulocyte-macrophage colony-stimulating factor (GM-CSF), a cytokine that accelerates the production of neutrophils, monocyte, and macrophages [41]. This drug increases neutrophil number and function in septic patients, but with no effect on overall survivals [42] even HLA-DR expression was restored. When a real defect in T cells number and function is documented, it will be better corrected using recombinant IL-7 or anti-PD-1 and -PDL-1-specific antibodies [43]. PD-1 and PDL-1 are widely expressed on immune effector cells, endothelial cells and bronchial epithelial cells during human sepsis [43]. Experimental data sound promising to improve survival in sepsis, at least in experimental models. IL-7, a fundamental molecule for T cell development and function [3], is another alternative in septic patients. IL-7 increases CD4 and CD8 T number and function of cells. One human trial on sepsis is on-going to validate the phase 2 before designing a phase 3 trial.

In conclusion, this study is the first looking at HLA-DR expression in blood and BM monocyte lineage in septic and non-septic ICU patients. It shows a pre-oriented downregulation of HLA-DR expression in the BM compartment before exiting to the blood. This BM immunosuppression is markedly increased in septic shock patients and does not result from an abnormal terminal differentiation of monocytes. The well-demonstrated blood mHLA-DR downregulation may also come from BM dysregulation. For granulocytes, if BM phenotype appears to be close to normal.

## Supporting Information

S1 Checklist STROBE Statement. Checklist of items that should be included in reports of observational studies.
(DOCX)

S1 Fig Expression of CD16 in BM monocyte lineage. Representative dot plots (patient 32) of CD16 expression in blasts, promonocytes and monocyte in BM.
(PPTX)

S1 Table Clinical characteristics of patients. Age, gender, main cause of presence in hospital and/or ICU, time of ICU admission, SAPS II, cause, time and conclusion of BM analysis. As indicated in first column: patients 1 to 11: septic shock in ICU, patients 12 to 19: sepsis in ICU, patients 20 to 33: non septic in ICU, patients 34 to 42: not in ICU.
(DOC)

S2 Table Differentiation stages of monocytic and granulocytic lineage in ICU patients BM. % in leucocytes (CD45+ cells). Comparison with results given by haematologist (microscopic analysis) (n = 11 except for Promyelo-myelo, Metamyelo and PMNs analyzed by FACs, n = 10).
(XLSX)

S3 Table Individual data. Blood and bone marrow Monocyte HLA-DR expression, leucocyte populations, HLA-DR and CCR2 expression in bone marrow monocyte lineage, CD11b and CD62L expression in bone marrow granulocyte lineage.
(DOC)
