# Supplementary material for: Downregulation of Blood Monocyte HLA-DR in ICU Patients Is Also Present in Bone Marrow Cells
Source: PLoS One. 2016 Nov 28;11(11):e0164489. doi: 10.1371/journal.pone.0164489 (PMC5125574; doi:10.1371/journal.pone.0164489)
Supplement: S1 Checklist — Checklist of items that should be included in reports of observational studies. (DOCX) [file pone.0164489.s001.docx]

STROBE Statement—checklist of items that should be included in reports of observational studies

|  | Item No. | Recommendation | Page  No. | Relevant text from manuscript |
| --- | --- | --- | --- | --- |
| **Title and abstract** | 1 | (*a*) Indicate the study’s design with a commonly used term in the title or the abstract | 1 | “blood monocyte HLA-DR” “ICU patients” “ bone marrow cells” |
|  |  | (*b*) Provide in the abstract an informative and balanced summary of what was done and what was found | 2 | “This study analysed the BM HLA-DR expression in intensive care patients (ICU) patients, which was compared to the BM monocytes of non-ICU patients and the blood monocytes of control healthy donors. In a subgroup of ICU patients, the phenotyping of the immature forms of monocytes and granulocytes in BM was also performed to explore potential dysfunctions in differentiation.” “The downregulation of monocyte HLA-DR occurs in both blood and BM of ICU patients without major changes in hematopoiesis” |
| Introduction | | | |  |
| Background/rationale | 2 | Explain the scientific background and rationale for the investigation being reported | 3 | A paradigm shift for sepsis involves the evolution of the blood immune status from a state of initial hyperactivity to early downregulation and immunodepression [1]. This immunodepression has been shown to involve both innate and adaptive immunity [2, 3] in circulating and tissue monocytes and lymphocytes [4]. “Among the potential parameters to monitor, blood monocyte HLA-DR was the most frequently monitored in the blood [5, 8, 9] and tissues [4]”. Although hematopoiesis has been investigated in hematological diseases and cancer in humans [18], it has not been described in intensive care patients with acute inflammation. |
| Objectives | 3 | State specific objectives, including any prespecified hypotheses | 3-4 | “Access to BM samples from in ICU patients gave us the opportunity to investigate the following aspects: 1- to evaluate the expression of HLA-DR in BM (marker of monocytic differentiation) and blood monocytes in ICU patients *versus* healthy controls and non-ICU patients; 2- to investigate CCR2 expression, which is known to increase from unspecialized cells to blood monocytes, and the expression of the receptor of MCP-1, which is the main chemokine responsible for monocytes exiting from BM; and 3- to quantify the CD11b and CD62L expression (adhesion molecules) in 4 stages of granulocyte differentiation in the myeloid lineage.” |
| Methods | | | |  |
| Study design | 4 | Present key elements of study design early in the paper | 4-6 | “We prospectively enrolled patients who were 18 years or older with systemic inflammation and/or sepsis, and the senior in charge made the decision to analyse the BM. The decision was motivated by inexplicably low counts in blood cell population(s) and was validated by an independent haematologist.”  “The specialist (internal medicine or rheumatology) determined whether a BM analysis should be conducted for non-ICU patients during medical consults or short hospitalizations, and such an analysis was used to evaluate the normal range of BM cells”  “the normal values for blood parameters were determined using blood samples from 28 healthy volunteers”  “Bone marrow analysis was blindly conducted by a trained haematologist.”  “Flow cytometry” (p5-6) |
| Setting | 5 | Describe the setting, locations, and relevant dates, including periods of recruitment, exposure, follow-up, and data collection | 1; 4 | “AP-HP, Hôpital Lariboisière, Department of Anesthesiology and Critical Care and SAMU, Paris, France”  “Between june 2007 and march 2014, we prospectively enrolled ICU patients”  “At the same time period, the specialist (internal medicine or rheumatology) determined whether a BM analysis should be conducted for non-ICU patients during medical consults or short hospitalizations, and such an analysis was used to evaluate the normal range of BM cells (conclusion made by a blinded haematologist) and the normal blood HLA-DR expression levels.” |
| Participants | 6 | (*a*) *Cohort study*—Give the eligibility criteria, and the sources and methods of selection of participants. Describe methods of follow-up  *Case-control study*—Give the eligibility criteria, and the sources and methods of case ascertainment and control selection. Give the rationale for the choice of cases and controls  *Cross-sectional study*—Give the eligibility criteria, and the sources and methods of selection of participants | 4 | “ICU patients: 18 years or older with systemic inflammation and/or sepsis, and the senior in charge made the decision to analyse the BM. The decision was motivated by inexplicably low counts in blood cell population(s) and was validated by an independent haematologist. BM analysis was aimed at diagnosing a macrophage activation syndrome, a toxin-induced BM dysfunction or a metabolic deficit. Patients who were pregnant, had myelodysplasia or were treated with chemotherapy and who had a risk of aplasia were excluded.”  “Non-ICU patients: At the same time period, the specialist (internal medicine or rheumatology) determined whether a BM analysis should be conducted for non-ICU patients during medical consults or short hospitalizations. When such BM analysis was normal (n = 9) (conclusion made by a blinded haematologist), the results were used to determine “normal” ranges of HLA-DR expression in BM monocyte. “Controls: the normal values for blood parameters were determined using blood samples from 28 healthy volunteers” |
|  |  | (*b*) *Cohort study*—For matched studies, give matching criteria and number of exposed and unexposed  *Case-control study*—For matched studies, give matching criteria and the number of controls per case | NA |  |
| Variables | 7 | Clearly define all outcomes, exposures, predictors, potential confounders, and effect modifiers. Give diagnostic criteria, if applicable | NA |  |
| Data sources/ measurement | 8* | For each variable of interest, give sources of data and details of methods of assessment (measurement). Describe comparability of assessment methods if there is more than one group | 4-5-6 | “Sepsis and septic shock were diagnosed and classified according to the ACCP/SCCM consensus criteria [19]”  “Bone marrow analysis was blindly conducted by a trained haematologist. Samples were obtained by sternal aspiration and were immediately smeared on glass slides. After May Grünwald Giemsa coloration, analysis was conducted under the microscope using 50 and 100X magnifications”  “Flow cytometry: The selection of cell populations was based on forward/side scattering and surface molecule expression. Blood monocytes were selected as CD14-positive (pos) cells with intermediate SSC for which HLA-DR expression was analysed. These blood monocytes were compared to bone marrow CD14 high cells, which were considered to be the most differentiated cells among the monocytic lineage [20]. For the BM monocyte lineage study, the following characteristics were used to differentiate between the different CD45-positive (marker of leucocytes) cell populations: myelo-/monoblasts: SSClow/CD34high; promonocytes: CD34neg/CD33high/CD14neg; monocytes: CD34neg/CD33high/CD14pos.  For the granulocyte lineage, the following characteristics were used to differentiate between the different CD45-positive cell populations: promyelocytes + myelocytes (promyelo-myelo) CD16low/CD66binterm-high/CD13high-low; metamyelocytes: CD16pos/CD66binterm/CD13interm and PMNs: CD16high/CD66blow/CD13high [20, 21].  For cell surface marker analysis, 50 µl of blood or bone marrow (EDTA Vacutainer, BD, Le Pont-de-Claix, France) was incubated with the appropriate antibodies: anti-CD14-FiTC, anti-CD3-FiTC (Beckman Coulter Immunotech, Marseille, France), anti-HLA-DR-PE, anti-CD62L-PE, anti-CD16-PerCP-Cy5.5, anti CD11b-PE (BD), anti-CCR2-PE (R&D Systems, Abingdon, UK), anti-CD33-PE-Cy7 (Novus Biologicals, Cambridge, UK), anti-CD34-APC (Miltenyi Biotec, Bergisch Gladbach, Germany), anti-CD13-APC (Invitrogen Molecular Probes, Camarillo, CA, USA), and anti-CD45-APC-Cy7 (Biolegend, San Diego, CA, USA).  The expression of HLA-DR, CCR2, CD11b and CD62L was quantified as the number of sites per cell (antibodies bound per cell, AB/C, Quantibrite™, BD). The normal range for blood mHLA-DR expression in healthy donors using our set-up conditions (n=28; median age, 34 yrs old (range, 18 – 57); sex ratio, 64% female) was 16884 (5842 - 29175) AB/C.”  “Statistical analysis: The results were expressed as the median and the interquartile range (IQR). Differences between patient groups and healthy donors were tested using the non-parametric Kruskal-Wallis test. When statistical significance was found, inter group differences were tested with the Mann-Whitney test. Differences between bone marrow and blood monocytes were tested using the non-parametric Wilcoxon test. Differences in marker expression between successive stages of differentiation in the granulocyte or monocyte lineage were tested using the non-parametric Friedman test. When statistical significance was found, the Wilcoxon test was used to make paired comparisons between stages.” |
| Bias | 9 | Describe any efforts to address potential sources of bias | 12-13 | “Contamination of the BM sample by blood monocytes should be ruled out to ensure that the BM HLA-DR^low^ monocytes that we observed are not from the blood. This technical limit can be excluded because the proportion of T cells (CD3+ cells, n=11) in BM samples was only 4.1%, which clearly differed from what was observed in the blood.” |
| Study size | 10 | Explain how the study size was arrived at | NA |  |

Continued on next page

| Quantitative variables | 11 | Explain how quantitative variables were handled in the analyses. If applicable, describe which groupings were chosen and why | 6; 9 | “The expression of HLA-DR, CCR2, CD11b and CD62L was quantified as the number of sites per cell (antibodies bound per cell, AB/C, Quantibrite™, BD).”  “The differentiation stages of monocytes and granulocytes were analysed either by FACs or microscopy (2S Table). Because of the various criteria used to discriminate between the different stages (protein expression for FACs and morphological elements using microscopy), some differences were observed. To limit these differences, cell populations were grouped according to the closest differentiation stages as follows: myeloblasts and monoblasts were pooled and analysed as the myelo-/monoblast stage, and promyelocytes and myelocytes were grouped into one promyelo-myelo population.” |
| --- | --- | --- | --- | --- |
| Statistical methods | 12 | (*a*) Describe all statistical methods, including those used to control for confounding | 6 | “Statistical analysis  The results were expressed as the median and the interquartile range (IQR). Differences between patient groups and healthy donors were tested using the non-parametric Kruskal-Wallis test. When statistical significance was found, inter group differences were tested with the Mann-Whitney test. Differences between bone marrow and blood monocytes were tested using the non-parametric Wilcoxon test. Differences in marker expression between successive stages of differentiation in the granulocyte or monocyte lineage were tested using the non-parametric Friedman test. When statistical significance was found, the Wilcoxon test was used to make paired comparisons between stages.” |
|  |  | (*b*) Describe any methods used to examine subgroups and interactions | NA |  |
|  |  | (*c*) Explain how missing data were addressed | S3 table | “missing value due to technical pb” |
|  |  | (*d*) *Cohort study*—If applicable, explain how loss to follow-up was addressed  *Case-control study*—If applicable, explain how matching of cases and controls was addressed  *Cross-sectional study*—If applicable, describe analytical methods taking account of sampling strategy | NA |  |
|  |  | (*e*) Describe any sensitivity analyses | NA |  |
| Results | | | | |
| Participants | 13* | (a) Report numbers of individuals at each stage of study—eg numbers potentially eligible, examined for eligibility, confirmed eligible, included in the study, completing follow-up, and analysed | 4; 6; 8 | “42 patients:   - ICU patients were either in septic shock (n = 11) or did not exhibit septic shock (n = 8 septic; n =14 non-septic) - Non ICU patients: n = 9   28 Healthy controls” |
|  |  | (b) Give reasons for non-participation at each stage | NA |  |
|  |  | (c) Consider use of a flow diagram | Figure 1 |  |
| Descriptive data | 14* | (a) Give characteristics of study participants (eg demographic, clinical, social) and information on exposures and potential confounders | 8; S1 table | Table 1 |
|  |  | (b) Indicate number of participants with missing data for each variable of interest | 9; S2 table; S3 table | Blood mHLA-DR was missing in 6 non-ICU patients.  Data from 1 patient are missing in BM granulocyte lineages analysis (ICU patients).  Data from 1 patient are missing for HLA-DR expression of monocyte differentiation stages (ICU patients). |
|  |  | (c) *Cohort study*—Summarise follow-up time (eg, average and total amount) | NA |  |
| Outcome data | 15* | *Cohort study*—Report numbers of outcome events or summary measures over time | NA |  |
|  |  | *Case-control study—*Report numbers in each exposure category, or summary measures of exposure | NA |  |
|  |  | *Cross-sectional study—*Report numbers of outcome events or summary measures | NA |  |
| Main results | 16 | (*a*) Give unadjusted estimates and, if applicable, confounder-adjusted estimates and their precision (eg, 95% confidence interval). Make clear which confounders were adjusted for and why they were included | NA |  |
|  |  | (*b*) Report category boundaries when continuous variables were categorized | NA |  |
|  |  | (*c*) If relevant, consider translating estimates of relative risk into absolute risk for a meaningful time period | NA |  |

Continued on next page

| Other analyses | 17 | Report other analyses done—eg analyses of subgroups and interactions, and sensitivity analyses | NA |  |
| --- | --- | --- | --- | --- |
| Discussion | | | | |
| Key results | 18 | Summarise key results with reference to study objectives | 12; 13 | “The main results of this study are as follows: 1- a similar downregulation of mHLA-DR in the blood and bone marrow in different groups of ICU patients compared to controls and non-ICU patients and 2- a dysfunction in the maturation of the granulocyte lineage in BM.”  “Our study also showed the stimulation of the granulocyte lineage in BM, which supports the peripheral recruitment of PMNs.” |
| Limitations | 19 | Discuss limitations of the study, taking into account sources of potential bias or imprecision. Discuss both direction and magnitude of any potential bias | 12 | Limits of the study: some limitations preclude the generalization of these results. The number of enrolled patients in ICU was relatively small (n =33). BM exploration was decided only when peripheral blood cell count was abnormal without clear etiologies. Consequently, the results on BM cannot be extended to patients not having abnormal blood count. Finally, the ICU patient inflammation etiologies were heterogeneous apart of severe sepsis or septic shock, including brain injuries, hemorrhagic or vascular diseases, major surgery and pre-eclampsia. This heterogeneity may also be seen as an advantage for investigation of life threatening situations for BM and blood cell phenotypes. |
| Interpretation | 20 | Give a cautious overall interpretation of results considering objectives, limitations, multiplicity of analyses, results from similar studies, and other relevant evidence | 12; 13; 14 | “In our study, HLA-DR expression in blasts was heterogeneous but was significantly higher than in mature CD14+ monocytes in BM. Because this level of HLA-DR expression (4910 [14419] sites per cell) in the blasts of ICU patients was not significantly different from the expression in mature BM monocytes in non-ICU patients (9218 [6392] sites per cell), this finding suggests a downregulation with differentiation in acute ICU situations. The progressive increase in CCR2 expression (the receptor to MCP-1) with BM monocyte differentiation also indicated that blood monocytes were mature and ready to respond to a MCP-1 gradient and exit BM towards the blood compartment [32]. These results suggest that HLA-DR downregulation in monocytes already occurs in BM, leading to an increase in HLA-DR^low^ blood monocytes. In addition, the expression of HLA-DR in blood monocytes was low, a fact that does not support the hypothesis that circulating cells prematurely exit the BM, which has a higher expression of HLA-DR (figure 3). Contamination of the BM sample by blood monocytes should be ruled out to ensure that the BM HLA-DR^low^ monocytes that we observed are not from the blood. This technical limit can be excluded because the proportion of T cells (CD3+ cells, n=11) in BM samples was only 4.1%, which clearly differed from what was observed in the blood.”  “Our study also confirmed the stimulation of the granulocyte lineage in BM, which supports the peripheral recruitment of PMNs. Under normal conditions, the BM neutrophilic granulocytes (CD15+, CD33+, CD43+, CD66b+) are composed of 7±4% of promyelocytes, 27±9.2% of myelocytes, 24±6.1% of metamyelocytes, and 42.2±12.8% of PMNs [34]. In our study, the promyelocytes and myelocytes maintained their capacity to proliferate, a property that is not normally present for metamyelocytes and mature PMNs [26]. The stimulation of proliferation in promyelocytes and myelocytes in our study is consistent with the observed increase of these cells in the peripheral blood in septic patients [35], which is independent of WBC total count, a feature that has the potential to predict outcome mainly during the 1^st^ week.”  “The expression of both CD11b and CD62L along the stages of differentiation in our study confirmed the findings in the literature and suggests that acutely inflamed conditions in patients in the ICU stimulates proliferation without any change in cell phenotypes in BM granulocytes.” |
| Generalisability | 21 | Discuss the generalisability (external validity) of the study results | 12 | The number of enrolled patients in ICU was relatively small (n =33). BM exploration was decided only when peripheral blood cell count was abnormal without clear etiologies. Consequently, the results on BM cannot be extended to patients not having abnormal blood count. Finally, the ICU patient inflammation etiologies were heterogeneous apart of severe sepsis or septic shock, including brain injuries, hemorrhagic or vascular diseases, major surgery and pre-eclampsia. This heterogeneity may also be seen as an advantage for investigation of life threatening situations for BM and blood cell phenotypes. |
| Other information | |  | | |
| Funding | 22 | Give the source of funding and the role of the funders for the present study and, if applicable, for the original study on which the present article is based | 15 | “This work was supported by a grant from the University Paris Diderot, Sorbonne Paris Cité.” |

*Give information separately for cases and controls in case-control studies and, if applicable, for exposed and unexposed groups in cohort and cross-sectional studies.

**Note:** An Explanation and Elaboration article discusses each checklist item and gives methodological background and published examples of transparent reporting. The STROBE checklist is best used in conjunction with this article (freely available on the Web sites of PLoS Medicine at http://www.plosmedicine.org/, Annals of Internal Medicine at http://www.annals.org/, and Epidemiology at http://www.epidem.com/). Information on the STROBE Initiative is available at www.strobe-statement.org.
