# Supplementary figures and images for: Downregulation of Blood Monocyte HLA-DR in ICU Patients Is Also Present in Bone Marrow Cells
Source: PLoS One. 2016 Nov 28;11(11):e0164489. doi: 10.1371/journal.pone.0164489 (PMC5125574; doi:10.1371/journal.pone.0164489)

## Slide 1
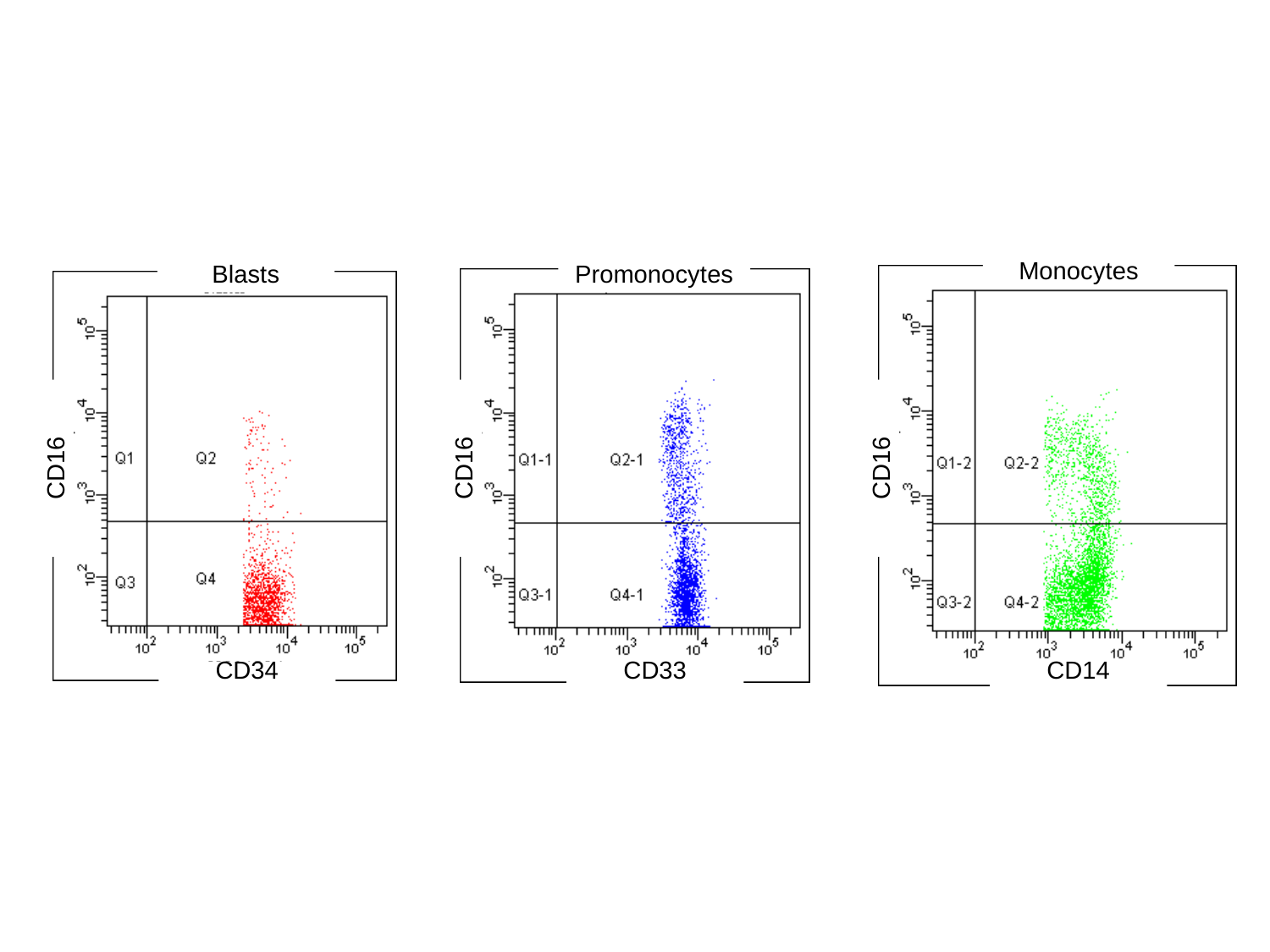

Monocytes
CD16
CD14
Blasts
CD16
CD34
Promonocytes
CD16
CD33

Supplement: S1 Fig — Representative dot plots (patient 32) of CD16 expression in blasts, promonocytes and monocyte in BM. (PPTX) [file pone.0164489.s002.pptx]
