# Supplementary material for: Downregulation of Blood Monocyte HLA-DR in ICU Patients Is Also Present in Bone Marrow Cells
Source: PLoS One. 2016 Nov 28;11(11):e0164489. doi: 10.1371/journal.pone.0164489 (PMC5125574; doi:10.1371/journal.pone.0164489)
Supplement: S1 Table — Age, gender, main cause of presence in hospital and/or ICU, time of ICU admission, SAPS II, cause, time and conclusion of BM analysis. As indicated in first column: patients 1 to 11: septic shock in ICU, patients 12 to 19: sepsis in ICU, patients 20 to 33: non septic in ICU, patients 34 to 42: not in ICU. (DOC) [file pone.0164489.s003.doc]

|  | N° | Age (y/o) | Sex | ICU | Main cause of hospitalization  / Main cause of ICU admission | Origin of sepsis | Indication for BM analysis | Time of ICU admission (days post hospital admission) | SAPS II at ICU admission | Time of BM analysis (days post ICU admission for ICU patients; post hospital admission for non-ICU patients) | Conclusion of BM analysis by hematologist |
| --- | --- | --- | --- | --- | --- | --- | --- | --- | --- | --- | --- |
| Septic shock ICU | 1 | 73 | M | yes | Peritonitis  / Septic shock | Abdomen | Thrombopenia, suspicion of Macrophage Activation Syndrome (MAS) | 0 | 77 | 5 and 18 | D5: probable MAS  D18: reactive BM,  Excess of promyelocytes, plasmocytes and macrophages,  Peripheral cause of thrombopenia |
|  | 2 | 83 | M | yes | Peritonitis  / Septic shock | Abdomen | Thrombopenia | 1 | 52 | 9 | Granulocytes lineage hyperplasia, Peripheral cause of thrombopenia |
|  | 3 | 62 | F | yes | Mesenteric ischemia  / Septic shock | Abdomen | Thrombopenia | 0 | 39 | 1 | Myelodysplastic syndrome probably related to sepsis,  Peripheral cause of thrombopenia |
|  | 4 | 48 | M | yes | Purpura fulminans / Septic shock & LV Failure | Blood | Thrombopenia | 0 | 54 | 5 | Reactive BM,  Peripheral cause of thrombopenia |
|  | 5 | 77 | M | yes | Severe bedsore / Septic shock | Bone | Thrombopenia, MAS suspicion | 1 | 44 | 7 | Granulocytes lineage augmentation, Peripheral cause of thrombopenia |
|  | 6 | 42 | M | yes | Respiratory failure / Septic shock | Lung | Leucopenia, lymphopenia | 0 | 57 | 2 | Interruption of granulocytes lineage at promyelocyte stage |
|  | 7 | 66 | F | yes | Intraperitoneal chemotherapy  / Septic shock | Abdomen | Thrombopenia | 11 | 61 | 8 | Reactive BM  Excess of promyelocytes and myelocytes,  Peripheral cause of thrombopenia |
|  | 8 | 75 | F | yes | Peritonitis  / Septic shock | Abdomen | Thrombopenia | 0 | 61 | 4 | Reactive BM,  Peripheral cause of thrombopenia |
|  | 9 | 78 | F | yes | Cardia adenocarcinoma, oesophagectomy  / Pneumopathy, respiratory distress | Lung | Thrombopenia | 1 | 35 | 12 | Reactive BM  Excess of promyelocytes,  Peripheral cause of thrombopenia |
|  | 10 | 48 | M | yes | Cardiac decompensation  / Cardiogenic shock, influenza, haemophilus pneumopathy | Lung | Thrombopenia | 2 | 45 | 19 | Reactive BM  MAS |
|  | 11 | 63 | F | yes | Persistent fever (Crohn’s disease) / Respiratory distress | Lung | Thrombopenia, MAS suspicion | 9 | 31 | 4 | Hemophagocytosis but no MAS,  Peripheral cause of thrombopenia |
| Sepsis ICU | 12 | 51 | F | yes | Acute colitis  / Total colectomy, allergic broncho-pulmonary aspergillosis | Lung | Innate immunity dysfunction, suspicion of chronic granulomatous disease | 24 | 21 | 34 | Reactive BM,  Excess of promyelocytes |
|  | 13 | 30 | M | yes | Cervical cellulitis | ENT | Neutropenia, lymphopenia | 0 | 37 | 2 | Erythroblastopenia |
|  | 14 | 44 | F | yes | Ileo-colic resection (Crohn’s disease)  / Post-operative peritonitis, septic myocarditis | Abdomen | Thrombopenia | 2 | 30 | 5 | Reactive BM,  Peripheral cause of thrombopenia |
|  | 15 | 43 | F | yes | Stroke  / Craniectomy, multiple infections | Lung, urinary tract, blood | Thrombopenia | 2 | 22 | 13 | Reactive BM,  Peripheral cause of thrombopenia |
|  | 16 | 67 | F | yes | Pyelonephritis  / Severe sepsis | Urinary tract | Anemia, thrombopenia | 1 | 39 | 2 | Reactive BM  Excess of granulocytes precursors,  Peripheral cause of thrombopenia |
|  | 17 | 41 | M | yes | Dyspnea  / Pneumopathy, severe hypoxemia | Lung | Lymphopenia | 0 | 19 | 4 | Normal BM  Excess of promyelocytes  Moderate plasmocytosis |
|  | 18 | 64 | M | yes | Total coloproctectomy (Crohn’s disease)  / Respiratory distress, peritonitis, MOF | Abdomen | Thrombopenia | 29 | 34 | 39 | Hyperplasia  Excess of promyelocytes,  Peripheral cause of thrombopenia |
|  | 19 | 56 | M | yes | Severe hypoxemic pneumopathy | Lung | Leukopenia | 0 | 23 | 15 | Normal BM |
|  | 20 | 51 | M | yes | Traumatic brain injury  / Craniectomy | Lung | Anemia, thrombopenia, lymphopenia | 1 | 24 | 12 | Reactive BM  Excess of promyelocytes,  Peripheral cause of thrombopenia |
|  | 21 | 19 | M | yes | Traumatic brain injury  / Subdural hematoma drainage, craniectomy | Lung | Thrombopenia | 0 | 18 | 8 | Reactive BM  Erythroid lineage abnormalities,  Peripheral cause of thrombopenia |
| Not septic ICU | 22 | 53 | F | yes | Digestive hemorrhage  / Hemorrhagic shock |  | Thrombopenia, MAS suspicion | 11 | 42 | 2 | Probable MAS |
|  | 23 | 85 | F | yes | Unsealed hip prosthesis  / Chronic hip infection, encephalopathy, renal failure |  | Thrombopenia | 21 | 50 | 3 | Reactive BM  Excess of monocytes and macrophages,  Mixed origin of thrombopenia (sepsis, bleeding, ribavirin) |
|  | 24 | 47 | M | yes | Cerebral hematoma  / Craniectomy, suspicion of kidney tumor |  | Multiple adenopathies | 1 | 47 | 5 | Normal BM |
|  | 25 | 24 | M | yes | Respiratory distress  / Acute pulmonary edema, mitral stenosis |  | Thrombopenia | 0 | 30 | 1 | Reactive BM  Excess of promyelocytes,  Peripheral cause of thrombopenia |
|  | 26 | 79 | M | yes | Cardiac left decompensation  / Hypoxemia, acute pulmonary edema, pulmonary infection |  | Anemia, thrombopenia | 3 | 28 | 36 | Normal BM,  Peripheral cause of thrombopenia |
|  | 27 | 73 | F | yes | Arthritis  / Suspicion of anaphylactic shock, MOF |  | Anemia | 31 | 53 | 4 | Decreased cell density  Excess of promyelocytes  Hemophagocytosis |
|  | 28 | 64 | F | yes | Bile duct carcinoma, duodenopancreatectomy  / Respiratory distress |  | Anemia, thrombopenia, PMN polynucleosis | 2 | 27 | 18 | Post radiation lesions but no signs of Hodgkin syndrome or myeloproliferative disease evolution |
|  | 29 | 66 | M | yes | Digestive hemorrhage  / Respiratory distress, hemodynamic unstability |  | Thrombopenia | 4 | 26 | 8 | Almost no megakaryocytes |
|  | 30 | 30 | F | yes | Pre-eclampsia, thrombopenia  / Uncontrolled hypertension |  | Thrombopenia | 6 | 6 | 3 | Normal BM,  Peripheral cause of thrombopenia |
|  | 31 | 58 | M | yes | Sinusitis  / Ethmoid biopsy, encephalitis |  | Thrombopenia, leukopenia | 60 | 37 | 3 | Reactive BM,  Excess of promyelocytes  MAS |
|  | 32 | 85 | F | yes | Acute cholecystitis  / Hyponatremia |  | Thrombopenia | 81 | 49 | 4 | Reactive BM,  Excess of promyelocytes,  Peripheral cause of thrombopenia |
|  | 33 | 63 | M | yes | Cerebral hematoma / Surgical drainage |  | Thrombopenia | 0 | 42 | 2 | Slight excess of immature granulous stages  Peripheral cause of thrombopenia |
| Not ICU | 34 | 75 | M | no | Medical consultation |  | Anemia |  |  | 0 | Suspicion of epithelial carcinoma, No abnormalities of hematopoietic lineages |
|  | 35 | 86 | M | no | Knee arthritis after septic shock |  | Increase of T NK cells proportion in blood |  |  | 112 | Slight excess of lymphocytes |
|  | 36 | 30 | F | no | Thrombo-embolic disease exploration |  | Thrombo-embolic disease exploration |  |  | 2 | Normal BM |
|  | 37 | 82 | F | no | Medical consultation |  | Anemia |  |  | 0 | Normal BM |
|  | 38 | 66 | M | no | Medical consultation |  | Thrombopenia |  |  | 0 | Normal megakaryocytes and granulocytes lineage,  Few signs of eythroblastic dysplasia |
|  | 39 | 61 | M | no | Health degradation, thoracic pain, walking difficulties |  | Gamma globulin peak in blood proteins |  |  | 12 | No myeloma |
|  | 40 | 72 | F | no | Health degradation |  | Anemia |  |  | 2 | Myeloma |
|  | 41 | 49 | M | no | Medical consultation |  | L2 plasmocytoma exploration |  |  | 0 | Normal BM |
|  | 42 | 81 | M | no | Respiratory distress |  | Thrombopenia, leukopenia |  |  | 3 | Normal BM |
