# Supplementary material for: Downregulation of Blood Monocyte HLA-DR in ICU Patients Is Also Present in Bone Marrow Cells
Source: PLoS One. 2016 Nov 28;11(11):e0164489. doi: 10.1371/journal.pone.0164489 (PMC5125574; doi:10.1371/journal.pone.0164489)
Supplement: S3 Table — Blood and bone marrow Monocyte HLA-DR expression, leucocyte populations, HLA-DR and CCR2 expression in bone marrow monocyte lineage, CD11b and CD62L expression in bone marrow granulocyte lineage. (DOC) [file pone.0164489.s005.doc]

|  | ICU patients | | | Non -ICU patients | |
| --- | --- | --- | --- | --- | --- |
|  | %  in leucocytes (FACs) | %  in leucocytes (microscope) | p  (FACs vs microscope, Wilcoxon) | %  in leucocytes (microscope) | p  (ICU vs  non ICU,  Mann Whitney) |
| Myelo/monoblasts | 0.283 [0.372] | 1.4 [1.075] | 0.0208 | 1.5 [1] | NS |
| Monocytes | 3.2 [4.325] | 4.3 [3.9] | NS | 3.9 [1.8] | NS |
| Promyelo-myelo | ~~28.3 [29.6]~~ 22.8 [24.7] | 18.8 [10.75] | ~~0.0367~~ NS | 16.0 [5.8] | NS |
| Metamyelo | ~~13.75 [8.2]~~ 21.55 [17.8] | 17.4 [6.625] | NS | 16.8 [2.9] | NS |
| PMNs | ~~17.95 [31.8]~~ 18.5 [32.4] | 36.2 [20.5] | NS | 36.8 [15.1] | NS |
